# Associations between structural injury and task-based corticomuscular connectivity after stroke

**DOI:** 10.3389/fneur.2025.1653349

**Published:** 2025-11-05

**Authors:** Rachana Gangwani, Jasper I. Mark, Benjamin Y. Huang, Jessica M. Cassidy

**Affiliations:** ^1^Human Movement Science, University of North Carolina at Chapel Hill, Chapel Hill, NC, United States; ^2^Department of Health Sciences, University of North Carolina at Chapel Hill, Chapel Hill, NC, United States; ^3^Department of Radiology, University of North Carolina at Chapel Hill, Chapel Hill, NC, United States

**Keywords:** stroke, structural injury, task-based corticomuscular connectivity, corticomuscular coherence (CMC), electroencephalography (EEG), electromyography

## Abstract

**Introduction:**

Stroke-related damage to structural pathways and functional connections disrupts neural network communication, contributing to behavioral deficits. A critical next step is to determine whether observed relationships between connectivity and behavior align with established neurobiological frameworks. This involves investigating structural-functional relationships as structural connectivity provides the scaffold for functional communication. Prior work primarily explored structural-functional relationships at rest, particularly between structural measures and cortico-cortical functional connectivity. However, because stroke impacts both cortical and muscular systems, incorporating task-based functional connectivity measurements that reflect synchronous activity between cortex and muscle may offer additional insight. Therefore, in this study, we examined relationships between structural injury and integrity measures with task-based functional connectivity between electrodes overlying sensorimotor cortical regions and affected upper-extremity musculature (referred to as corticomuscular coherence; CMC).

**Methods:**

Individuals with early subacute stroke admitted to an inpatient rehabilitation facility completed simultaneous electroencephalography (EEG) and electromyography (EMG) recordings during a grip task. Corticospinal tract (CST) injury and integrity were computed from structural and diffusion-weighted imaging. CMC measurements involving electrodes overlying ipsilesional motor areas and affected upper-extremity musculature were computed in frequency bands relevant to neural injury (delta, 1–3 Hz) and motor function (low beta, 13–19 Hz; high beta, 20–30 Hz). Correlational analyses were performed to ascertain relationships between structural and coherence measurements. To account for inter-individual heterogeneity, analyses were repeated for CST injury and integrity subgroups.

**Results:**

Of the 30 individuals enrolled, EEG data from 21 individuals who were able to complete the grip task were analyzed (10 females; 67.9 ± 9.8 years; 11.3 ± 4.1 days post-stroke). No significant structure-function associations were observed across the group. However, in the mild-moderate CST injury subgroup (*n* = 11), greater injury correlated with higher coherence between electrodes overlying the supplementary motor area and affected extensor digitorum (high beta: ρ = 0.83, *p* = 0.001). Similarly, in the subgroup depicting higher CST integrity (*n* = 9), CST integrity positively related to coherence between electrodes overlying the ipsilesional primary motor cortex and affected biceps (low beta: *r* = 0.94, *p* = 0.0001).

**Discussion:**

Findings exclusive to CST injury/integrity subgroups underscore the complexity of structure-function relationships in stroke. Associations between CMC measures in motor-relevant frequency bands with measures reflecting CST microstructure suggest that post-stroke structural injury modulates task-based corticomuscular connectivity. The identification of specific cortical regions and muscles depicts varying adaptive and/or compensatory neuroplastic-like mechanisms, providing mechanistic insights that could inform targeted rehabilitation strategies to optimize post-stroke recovery.

## Introduction

Stroke is characterized by disrupted structural and functional connectivity, impacting neuronal communication in regions both local to and remote from the immediate injury (1, 2). Disordered connectivity influences post-stroke recovery and contributes to impairments across multiple domains, including motor, speech, and cognition (3, 4). As much of the existing evidence predominantly involves correlational analyses between connectivity and behavior, advancing toward a mechanistic understanding of these relationships is essential for elucidating stroke recovery mechanisms (5). One approach to achieving this is by investigating the relationships between structural and functional connectivity (5).

The impetus for examining structure-function relationships is driven by the principle that the brain operates through an intricate framework of neurons and their interconnected pathways (6, 7). It is postulated that structural connectivity serves as the scaffolding for functional connectivity (8). Previous work investigating structure-function relationships demonstrated that structural damage to the corticospinal tract (CST) is associated with disordered somatomotor network connectivity and poorer motor status post-stroke (9, 10). Notably, the assessment of connectivity in these studies involved neuroimaging and/or neurophysiological recordings acquired at rest. While resting-state neural activity serves as the basis for ongoing brain function and network organization (11), examining structure-function relationships during task performance affords additional insight (12). Recent work reported that 38% of functional connections differ between task-oriented and resting states (13), which suggests a reconfiguration in functional connections during activity. Such reconfigurations to accommodate task demands are likely impacted post-stroke (14). Moreover, prior structure-function studies largely focused on associations between structural measures and cortico-cortical connectivity. Because stroke impacts both cortical and muscular systems, examining functional connectivity between sensorimotor regions and upper-extremity musculature may provide additional insight into post-stroke motor recovery.

In this study, we determined associations between structural MRI-derived measures and functional connections between brain and muscle sources from task-based electroencephalography (EEG) and electromyography (EMG) recordings, known as corticomuscular coherence (CMC). As an index of functional connectivity, CMC represents synchronization between cortical and muscle activity via the CST (15). Prior work demonstrated that individuals post-stroke exhibited reduced CMC in a motor-relevant frequency band (beta, 13–30 Hz) during upper-extremity movement compared to unimpaired controls (16–18). Others have shown that reduced beta-band CMC correlated with poorer motor function (17, 19), and increases in beta CMC paralleled motor recovery (17, 20). We computed CMC between EEG/EMG electrodes overlying primary and secondary motor regions and upper-extremity musculature in frequency bands related to sensorimotor function [low beta (13–19 Hz) and high beta (20–30 Hz)] (21) and low frequency delta (1–3 Hz) oscillations, which have been previously associated with post-stroke neural injury and motor status (22). To comprehensively assess structural connectivity, we measured both CST injury and integrity, recognizing the distinction between tract quantity (injury reflects the extent of damage) and quality (integrity reflects the functionality of the remaining tract) (23).

As CMC signifies communication via the CST, we hypothesized that greater CST injury would reduce the cortical drive to muscle, thereby yielding lower CMC values. Conversely, greater CST integrity would positively correlate with CMC. Recognizing that variability in stroke-related injury across individuals may obscure specific structure-function associations (24), we investigated these relationships in subgroups stratified by CST injury and integrity. We anticipated that these subgroups would reveal stronger structure-function associations.

## Methods

### Participants

We recruited individuals aged 18 years and older with stroke hospitalized in an inpatient rehabilitation facility (IRF). Exclusion criteria included a major, active, neurological, or psychiatric conditions, diagnosis apart from stroke affecting paretic extremity function, deficits in communication or English proficiency, concurrent enrollment in an interventional study, MRI or EEG contraindications, prior stroke history, and bilateral hemisphere involvement. Participants provided written consent approved by the Institutional Review Board at the University of North Carolina, Chapel Hill. Participants completed an MRI scan, EEG and EMG recordings, and behavioral assessments upon enrollment.

### EEG and EMG acquisition

**Figure 1:**
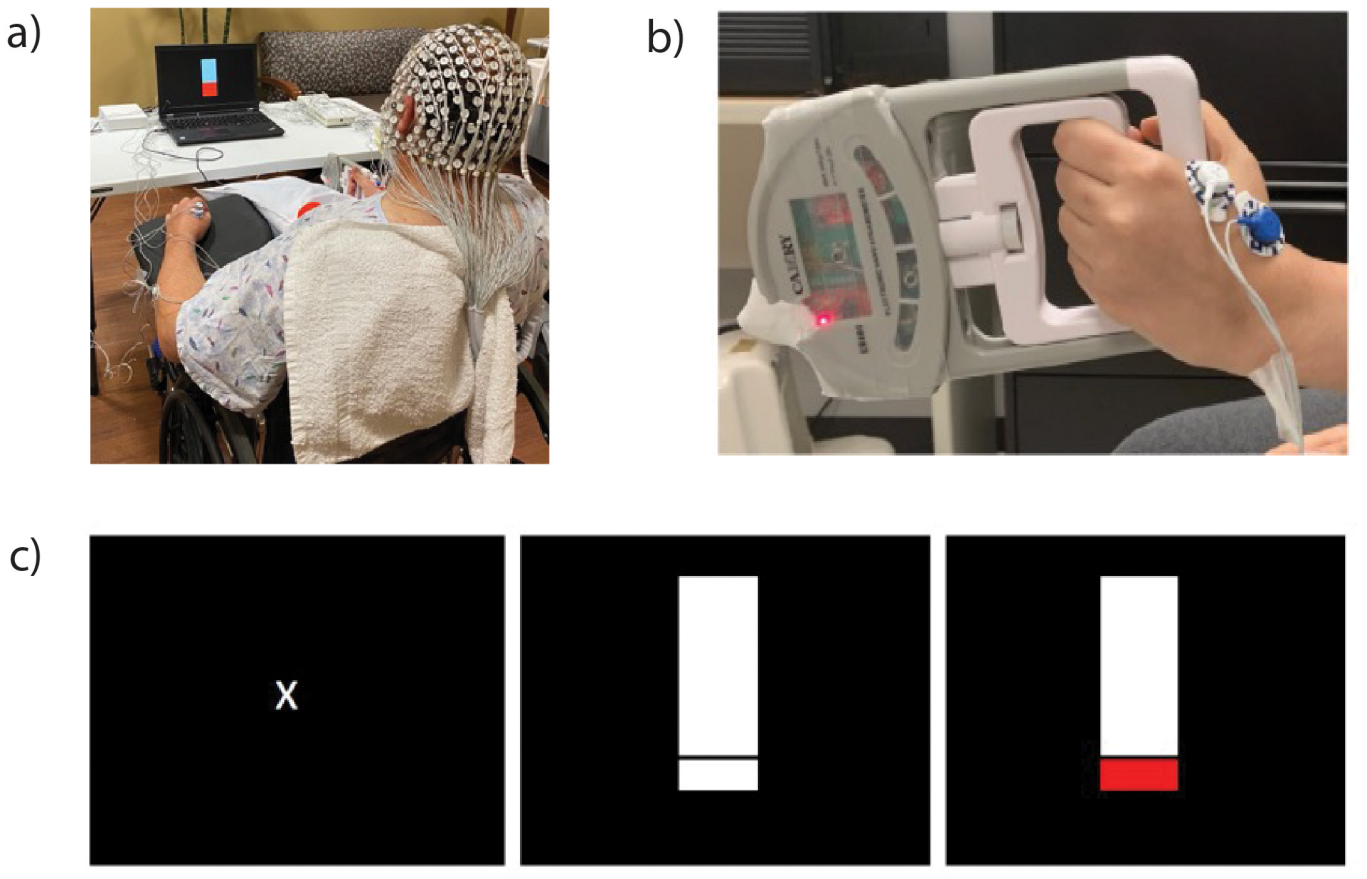
Participants wore an electroencephalography (EEG) net and electromyography (EMG) electrodes on bilateral upper-extremity muscles **(a)**. Participants squeezed a grip device **(b)** when the laptop images changed from rest [**(c)**, left] to a thermometer [**(c)**, center] and raised the thermometer to a target that corresponded to 20% of their maximal voluntary isometric force [**(c)**, right].

Participants donned a 256-electrode EEG net (Electrical Geodesics, Inc., Eugene, OR) and surface EMG electrodes on bilateral extensor and flexor digitorum, first dorsal interossei (FDI), and biceps brachii muscles (Figure 1a). Data were collected at a sampling rate of 1,000 Hz using a high input impedance Net Amp 400 amplifier and Net Station 5.4.2 software. Synchronization of EEG and EMG activity was accomplished using a Physio16 box and Cedrus StimTracker system (Cedrus Corp., San Pedro, CA).

During EEG and EMG recordings, participants performed a goal-directed submaximal isometric grip task (Figure 1b). This task was selected because of its functional relevance to activities of daily living and because of its feasibility early post-stroke compared to fine motor tasks. Participants were randomized to perform the task with their affected extremity first followed by their less-affected extremity. Before the recording, we determined participants' average maximal voluntary isometric force for each extremity across three trials. We determined 20% of their average maximal voluntary isometric force, which served as their target during task performance (Figure 1c). We chose a 20% value to mitigate fatigue arising from multiple trials and prior work indicating that typical force levels involved in squeezing/grip correspond to 20%−50% of maximal force output (25). Participants performed five practice trials before completing two blocks of 20 trials per extremity with a 1-min break between blocks. Each trial lasted 5 s with inter-trial intervals ranging from 7 to 15 s. Procedures were repeated on the opposite side following a brief break where investigators checked EEG and EMG electrode placement and signal quality. Participants maintained a sitting position with their forearm and wrist positioned to accommodate task requirements.

### EEG and EMG pre-processing

Raw data were exported to Matlab 2017b (Mathworks, Natick, MA) for processing using EEGLAB (26). Consistent with published protocols (22, 27), data were bandpass filtered between 0.5 and 50 Hz, and electrodes overlying cheek and neck regions were removed (194 electrodes remaining). Data were re-referenced before segmentation into 1-s non-overlapping epochs. Data underwent visual inspection and an Infomax independent components analysis to remove artifacts (28), and a spatial Laplacian filter was applied. Similarly, EMG data were transferred to MATLAB for offline processing that involved bandpass filtering from 10–50 Hz to 70–100 Hz. The unrectified data were passed through a Hilbert transform to obtain the signal envelope before rectification. EEG and EMG data were concatenated with CMC trial windows defined as 1,000 ms before and 4,000 ms after stimulus onset. Data were flipped so that the left hemisphere and right upper-extremity corresponded to the ipsilesional hemisphere and affected extremity for all participants.

### EEG and EMG data measures

CMC values are reported as the analog of the squared correlation coefficient between two distinct signals from EEG and EMG leads. Specifically, coherence is calculated with respect to the cross-spectrum density of signals S1 and S2, for each frequency of interest (f) [P_S1, S2_(f)] and the auto-spectrum densities of S1 and S2 for each frequency of interest [P_S1_(f) and P_S2_(f)] as noted in the following equation (29, 30).


$$\begin{array}{l} Coh_{S1,S2}\left(f\right)=\frac{{\left|P_{S1,S2}\left(f\right)\right|}^{2}}{\left|P_{S1}\left(f\right)|\times |P_{S2}\left(f\right)\right|} \end{array}$$

CMC measurements were computed between electrodes overlying the ipsilesional primary motor cortex (M1) and secondary motor areas including the premotor cortex, supplementary motor area (SMA), and parietal regions with each affected extremity upper-extremity muscle. We selected M1 based on work demonstrating reduced CMC between M1 and upper-extremity musculature following stroke (18, 19). We included secondary motor areas due to their role in motor control, visuomotor processing, and planning, organizing, and execution of goal-directed behavior (31, 32), important components of the task employed in this work. Table 1 lists the corresponding electrodes for each region of interest. We recorded EMG activity from electrodes overlying flexor digitorum and FDI (agonists) which are responsible for generating and stabilizing the grip force, and from extensor digitorum and biceps (antagonists), which prevent excessive finger flexion and maintain forearm and elbow position. We computed delta CMC since previous work identified that resting-state delta-band coherence with ipsilesional M1 correlated to greater injury and poor motor status in the sub-acute stroke stage (22). Additionally, other work reported reduced delta CMC between the cortex and thigh and leg muscles in individuals with chronic stroke compared to controls during a standing balance task that involved swaying the support surface (33). Additionally, we measured beta CMC given the prominence of beta oscillations during isometric muscle contraction (34), its involvement in motor control (21, 35), and evidence that demonstrated diminished beta CMC post-stroke (16, 19). We evaluated both low (13–19 Hz) and high (20–30 Hz) beta CMC, as recent studies have demonstrated distinct neural sources and roles in motor control between these frequency bands (36, 37). For each region of interest (ROI), CMC values were calculated as the average coherence across all EEG–EMG channel pairs overlying the ROI and the corresponding muscle. Coherence values range from 0 to 1 where 1 indicates consistent amplitude ratios and phase differences between signals across time in a given frequency band.

**Table 1:** Regions of interest.

**Region**	**Ipsilesional/Left electrodes**	**Contralesional/Right electrodes**
PMC	E30, E36, E41, E42, E43, E49, E50, E56, E57	E197, E204, E205, E206, E212, E213, E214, E215, E224
M1	E51, E52, E58, E59, E60, E65, E66	E155, E164, E182, E183, 184, E195, E196
Pr	E76, E77, E85, E86, E87, E88, E89, E96, E97, E98, E99, E100, E106, E107, E108, E109, E110, E118	E127, E128, E129, E130, E140, E141, E142, E151, E152, E153, E160, E161, E162, E163, E169, E170, E171, E172
SMA	E6, E7, E8, E15, E16, E17, E23, E24, E198, E207	

PMC, premotor cortex; M1, primary motor cortex; Pr, parietal cortex; SMA, supplementary motor area.

### Structural imaging and injury quantification

Structural MRIs were acquired on a 3-Tesla Siemens TrioTim Syngo or Skyra scanner or a 1.5-Tesla Siemens Aera scanner and included a high-resolution T1-weighted scan, a T2-weighted fluid-attenuated inversion recovery sequence, and diffusion tensor imaging. The protocol and specific parameters are described elsewhere (27). Using validated methods, we computed lesion volume and percent CST injury (23, 38) with higher values indicating greater magnitude of *tract injury*. We also calculated fractional anisotropy (FA) values of the CST at the level of the cerebral peduncle (23) with higher values indicating greater *tract integrity*. Considering inter-individual heterogeneity in stroke-related injury and recovery mechanisms (39), and work that recommends defining homogenous subgroups to better understand neural reorganization and recovery post-stroke (24), we stratified the cohort into subgroups based on the median values of percent CST injury and integrity.

### Statistical analysis

Statistical analyses were completed in JMP Pro 17 (SAS, Cary, NC). Correlations were performed to determine associations between structural and functional measures across the cohort. Parametric statistics were applied to data that were normally distributed data (confirmed by the Shapiro–Wilk test) or normalized following log transformation. Correlations were repeated for injury and integrity subgroups. To address multiple comparisons in CMC analyses (4 muscles × 3 frequency bands × 4 cortical regions), we employed a false discovery rate (FDR) correction using the Benjamini–Hochberg procedure (40) with *p* < 0.05 indicating significance. Exploratory analyses included determining associations between significant CMC measures and assessments of upper-extremity function (Upper Extremity Fugl-Meyer; UEFM) and performance (Action Research Arm Test; ARAT).

## Results

This work features a cohort published in a series of inpatient stroke studies (27, 41). Of the 30 individuals enrolled, 21 participants were able to complete the grip task with their affected extremity (10 females; mean age 67.9 ± 9.8; 11.3 ± 4.1 days post-stroke; Table 2). Nine participants had severe hemiparesis (UEFM range: 2–12 points out of 66), preventing task performance or adequate force generation for detection by the grip device. Twenty-one participants were included in analyses involving CMC and CST injury while 17 participants were included in analyses involving CMC and CST integrity.

**Table 2:** Participant demographics and assessment scores (*n* = 21).

**Descriptor**	**Sample size**	**Mean (SD) Median [IQR]**	**Range**
**Sex**			
Female	10	–	–
Male	11	–	–
**Race**			
Black	5	–	–
White	16	–	–
Age (years)		67.9 (9.8)	51–85
**Stroke type**			
Hemorrhagic	2	–	–
Ischemic	19	–	–
**Lesioned hemisphere**			
Left	8	–	–
Right	13	–	–
Days post-stroke		11.3 (4.1)	5–19
Length of IRF stay (days)		13.2 (5.1)	4–25
NIHSS (max = 42)		2 [1–3]	0–7
Lesion Volume (cc)		6.8 (10.5)	0.08–36.08
Percent CST injury		48.5 (40.4)	0–100
Mild-moderate	11	13.6 (19.3)	0–50
Severe	10	86.8 (9.9)	75–100
FA CST		0.6 (0.08)	0.37–0.77
High	9	0.6 (0.04)	0.62–0.77
Low	8	0.5 (0.07)	0.37–0.61
UEFM (max = 66)		56 (9.9)	33–66
Mild-moderate injury	11	60.5 (5.8)	49–66
Severe injury	10	51.2 (11.4)	33–65
High CST integrity	9	56.5 (10)	37–66
Low CST integrity	8	53.7 (12)	33–66
ARAT (max = 57)		47 (10.6)	26–57
Mild-moderate injury	11	51 (9.7)	28–57
Severe injury	10	42.7 (10.2)	26–55
High CST integrity	9	49 (10.7)	28–57
Low CST integrity	8	44 (12)	26–57
MoCA (max = 30)		22.5 (4.3)	14–29

Values presented as mean and standard deviation (SD) or median and interquartile range (IQR).

IRF, inpatient rehabilitation facility; cc, cubic centimeters; NIHSS, National Institutes of Health Stroke Scale; CST, corticospinal tract; FA, fractional anisotropy; UEFM, Upper Extremity Fugl-Meyer; ARAT, Action Research Arm Test; MoCA, Montreal Cognitive Assessment.

Using the median value for percent CST injury, individuals were classified into mild-moderate injury ( ≤ 50% injury) or severe subgroups (>50% injury). Similarly, based on FA values, we classified individuals into high-integrity (FA ≥ 0.62) or low-integrity subgroups (FA <0.62). Stroke-related injury constructed from lesion maps for the CST injury subgroups is depicted in Supplementary Figure S1.

**Figure 2:**
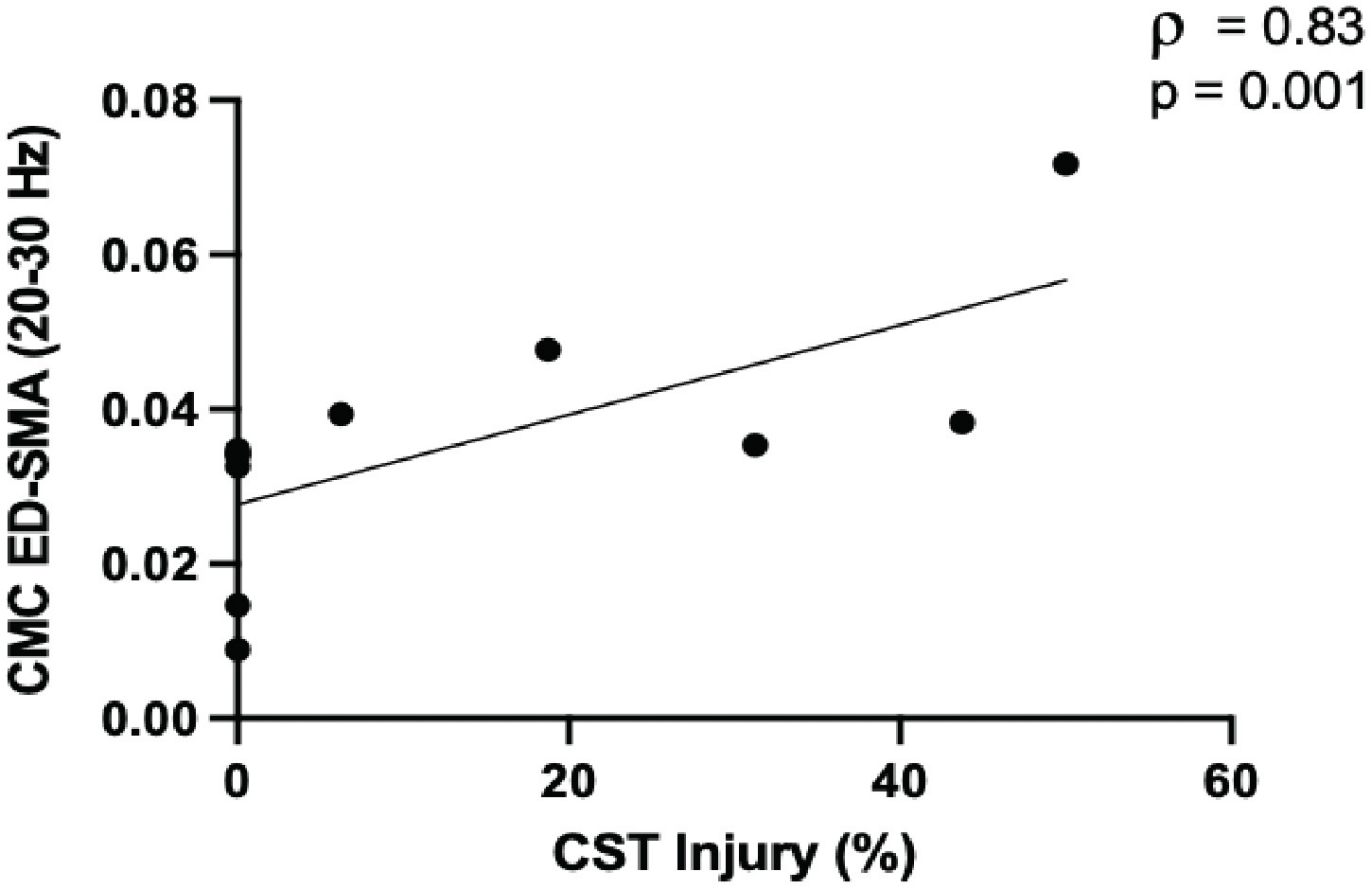
Greater corticospinal tract (CST) injury in the mild-moderate subgroup ( ≤ 50%, *n* = 11) was positively associated with high beta CMC between electrodes overlying the supplementary motor area (SMA) and extensor digitorum (ED).

We identified several associations between CMC and CST injury across the cohort (*n* = 21) and in subgroups before FDR correction (Supplementary Table S1). Following FDR correction, high beta CMC between electrodes overlying SMA and affected extensor digitorum positively related to CST injury in the mild-moderate injury subgroup (ρ = 0.83, *p* = 0.001; Figure 2).

**Figure 3:**
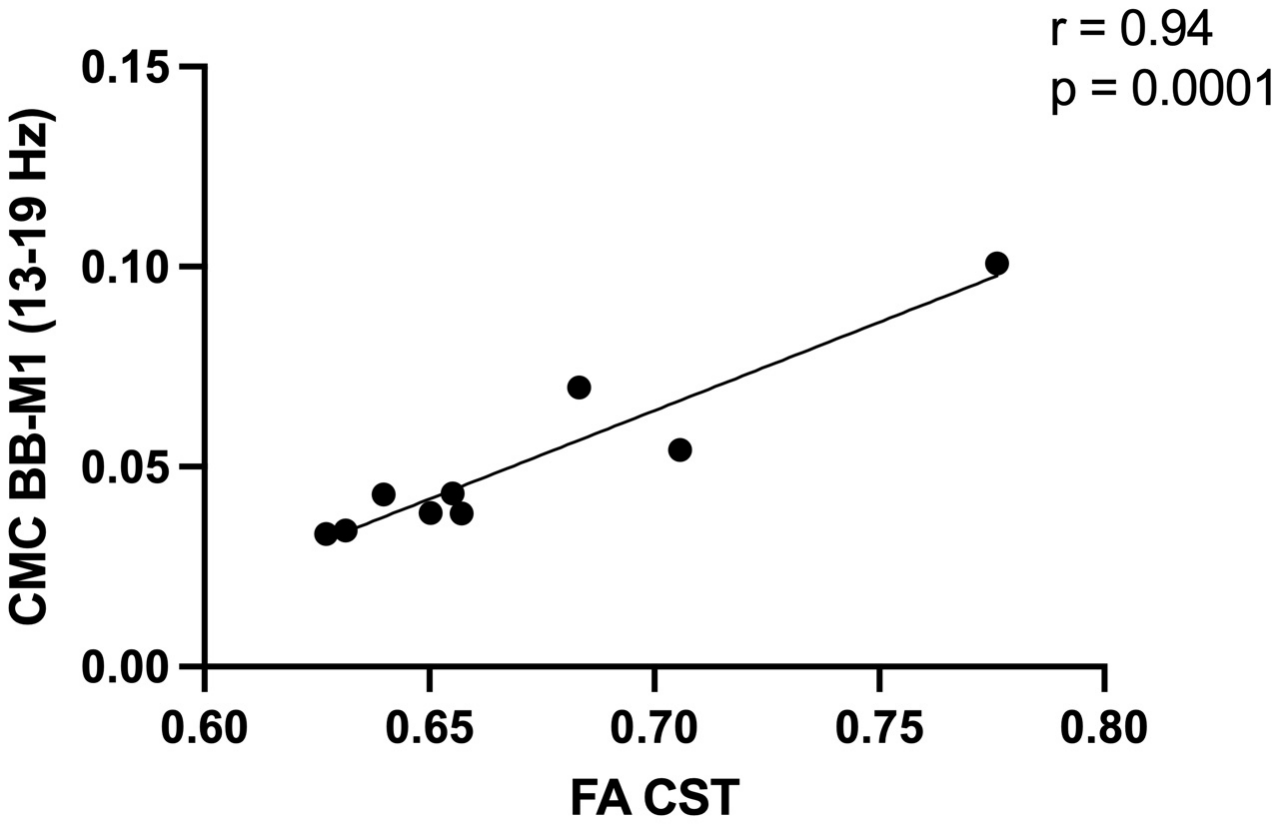
Greater corticospinal tract (CST) integrity in the high integrity group [fractional anisotropy (FA) ≥0.62, *n* = 9] was positively associated with low beta corticomuscular coherence (CMC) between electrodes overlying the primary motor cortex (M1) and biceps brachii (BB).

We did not observe associations between CMC and CST integrity across the cohort (*n* = 17; ρ range = 0.006–0.58, *p* range = 0.07–0.98). In the high-integrity group (*n* = 9), several CMC values were associated with CST integrity before FDR correction (Supplementary Table S2). Following FDR correction, low beta CMC between electrodes overlying ipsilesional M1 and affected biceps positively correlated with CST integrity (*r* = 0.94, *p* = 0.0001; Figure 3). We did not find any association between CMC values and CST integrity in the severe group (*n* = 8; ρ range = 0.009–0.57, *p* range = 0.06–0.97). To justify the reliability of these reported associations (i.e., our ability to detect these associations), we performed a *post hoc* power analysis using the observed correlation coefficients and current sample sizes. The achieved power was 0.94 (CST injury and high beta CMC between electrodes overlying SMA-extensor digitorum) and 0.99 (CST integrity and low beta CMC between electrodes overlying M1-biceps).

To rule out confounding effects of EEG power, we conducted *post hoc* analyses replacing beta coherence with power which revealed no significant correlations (ρ = 0.02, *p* = 0.93). Similarly, we assessed the impact of stroke lesion volume on CST injury, integrity, and CMC values. We found no significant associations between stroke volume and CST injury (ρ = −0.12, *p* = 0.70) and integrity (ρ = 0.23, *p* = 0.57), nor with beta CMC values (SMA-extensor digitorum: ρ = 0.30, *p* = 0.37; M1-biceps: ρ = 0.34, *p* = 0.29) that were identified as significant in subgroup analyses.

We conducted *post hoc* analyses to explore structure-function associations in those with severe injury, entailing ipsilateral functional connections between electrodes overlying the contralesional hemisphere and affected extremity to account for the possibility of increased recruitment of contralesional substrate(s) in individuals with severe injury (42). We initially found that greater CST injury was associated with reduced high beta CMC between electrodes overlying contralesional parietal and FDI (ρ = −0.67, *p* = 0.03), but this finding was not present after FDR correction.

Exploratory analyses in the full cohort (*n* = 21) indicated no significant associations between high beta CMC between electrodes overlying the SMA and extensor digitorum with either UEFM (ρ = −0.33, *p* = 0.13) or ARAT (ρ = −0.17, *p* = 0.44). Similarly, low beta CMC between electrodes overlying the M1 and the biceps did not correlate with either UEFM (ρ = 0.18, *p* = 0.43) or ARAT (ρ = 0.28, *p* = 0.21). Associations between CMC and motor assessments within injury and integrity subgroups are reported in Supplementary Table S3.

## Discussion

This study investigated relationships between structural measures and task-based corticomuscular connectivity in individuals with early subacute stroke. Unlike previous research that examined resting-state neural connectivity, our study involved assessing task-based corticomuscular connectivity alongside *both* structural injury and integrity measures. We hypothesized that CST damage or reduced CST integrity would negatively impact task-based brain-muscle connectivity. Interestingly, our findings revealed a nuanced account, demonstrating positive correlations between both CST injury and integrity with beta CMC, particularly in those with lower CST injury and higher tract integrity. These results underscore the intricate and complex nature of structural-functional connectivity dynamics post-stroke.

In our cohort which exhibited considerable heterogeneity, we observed no significant structure-function associations. However, when the cohort was stratified, significant associations emerged. In the mild-moderate CST injury subgroup, greater injury correlated positively with beta CMC between electrodes overlying SMA and extensor digitorum. In individuals with greater CST integrity, we observed greater beta CMC between electrodes overlying M1 and biceps. The presence of significant associations in more homogenous subgroups aligns with our hypothesis but diverges from others that reported no association between CST integrity and CMC (19). This group employed a wrist extension task while we utilized a goal-directed isometric grip task which likely introduced additional demands related to motor planning and real-time adjustment to grip. This group also assessed CST integrity at the level of the posterior limb of internal capsule which includes fibers from corticopontine and corticorubral tracts (43). In contrast, we measured at the level of the cerebral peduncle, where CST fibers are more concentrated (44), potentially increasing the sensitivity to detect microstructural alterations. These methodological differences likely contributed to the discrepancy in findings.

The relationships between CST injury and integrity with specific functional connections provide insights into potential mechanisms of adaptive plasticity post-stroke. In neurotypicals performing a grip task, the motor cortex exhibits phase coupling with contralateral muscles, driving beta CMC (34). Thus, we expected increased coherence between electrodes overlying M1 and primary grip muscles such as the FDI and flexor digitorum, and less coupling between electrodes overlying M1 and biceps, which stabilizes and maintains arm positioning during the grip task. However, our findings indicate a potential shift in motor control strategies as individuals with higher CST integrity exhibited greater CMC between electrodes overlying M1 and biceps, suggesting potential adjustments by the motor cortex and biceps to ensure proximal control for grip stability. This is consistent with neuromuscular compensation strategies where reliance on proximal stability compensates for weaker distal control. As proximal control is a pre-requisite for precise distal functions like grip (45), the observed CMC between electrodes overlying M1 and biceps may be relevant early post-stroke.

The unexpected positive association between CST injury and CMC between electrodes overlying SMA and extensor digitorum may reflect compensatory recruitment of alternative pathways. However, this CMC between electrodes overlying SMA and extensor digitorum was also observed in individuals without CST injury (0% lesion overlap). We surmise that task-specific recruitment of the SMA explains this positive association. Although not directly involved in movement execution, the SMA is integral in maintaining grip force, timing, and monitoring ongoing tasks (31)—essential components of successful isometric grip performance. Relatedly, the extensor digitorum which works synergistically with the flexor digitorum to maintain grip (46) may be recruited to preserve motor function. Thus, increased CMC between electrodes overlying SMA and extensor digitorum may reflect both task-specific recruitment and compensatory response to CST damage. While higher CST integrity may support efficient M1-to-muscle communication, enabling M1 to fulfill its role in motor execution, greater CST injury may require compensatory reliance on SMA to sustain motor function (47). These distinct brain-muscle connections could inform the development of targeted treatment strategies to restore disrupted connectivity and optimize recovery.

It is important to note that CMC between electrodes overlying the motor areas and FDI muscle did not survive FDR correction. Although our *post hoc* power analysis substantiated the adequacy of our current sample sizes, a larger sample in future work may provide greater power to detect additional associations, especially in those muscles more directly engaged in grip. Relatedly, larger-scale longitudinal studies would enable additional examination to determine whether these functional connections shift toward distal muscles during later recovery stages based on the extent of structural injury. Finally, the absence of significant associations involving delta CMC might be attributed to the task employed in this study. Beta-band CMC (≈15–30 Hz) is the canonical rhythm observed during steady, isometric contractions and reflects corticospinal drive. As this study incorporated an isometric grip task, beta CMC was potentially more robust to CST and sensorimotor system function in comparison to delta oscillatory activity.

The significant associations observed in the mild-moderate CST injury and high CST integrity subgroups suggest a threshold-like effect. The greater beta CMC in these subgroups may imply that these individuals possess a sufficient degree of corticomuscular connectivity that facilitates effective communication between cortical areas and motor units. In contrast, the absence of associations in severe CST injury and low CST integrity subgroups may indicate neural adaptations not captured by CMC involving electrodes overlying the ipsilesional cortical substrates. Interestingly, *post hoc* analyses exploring contributions to CMC from electrodes overlying the contralesional hemisphere revealed no associations. This finding may be attributed to the subacute stage of our participants, as ipsilateral functional connectivity may not fully engage until later stages of recovery. Moreover, individuals with severe CST injury may rely on alternative pathways like the reticulospinal tract (48). Altogether, these findings support the use of subgroup analyses to mitigate variability across individuals with stroke in an effort to further elucidate mechanisms related to injury severity (24). Identifying thresholds of corticomuscular connectivity related to the degree of CST preservation may present potential clinical utility. The ability to monitor changes in corticomuscular connectivity thresholds, for instance, may inform the timing and intensity of therapeutic interventions. Patients above the threshold may benefit from strategies that reinforce existing corticospinal pathways, whereas those below the threshold may benefit from treatment approaches that engage compensatory circuits or alternative motor pathways. Monitoring changes in CMC relative to this threshold could therefore serve as a valuable biomarker for tailoring and evaluating rehabilitation strategies. Because we observed corticomuscular associations specific to low and high beta frequency bands, the examination of frequency-specific thresholds for various corticomuscular circuits may further differentiate the role of low and high beta oscillatory activity in stroke recovery.

We acknowledge limitations of this study. First, our analyses excluded participants with severe hemiparesis who were unable to perform the grip task. This highlights both the limitations of the task and the grip device's sensitivity to detect muscle force. Second, we did not exclude participants based on lesion location or the extent of damage to cortical regions of interest. While this enhances generalizability, we acknowledge the impact of these factors on EEG signal interpretation and observed connectivity patterns. Third, our cohort of 21 participants exhibited a bimodal distribution, a common observation in studies focusing on the subacute recovery phase (24), with participants experiencing mild-moderate CST injury (0%−50%) or severe injury (>75%), but no participants in the 50%−75% range. As such, our findings should be interpreted in the context of these sample characteristics including stroke stage, CST damage, and motor impairment level. Lastly, the absence of significant associations between motor status and CMC measures may reflect limited statistical power due to the modest sample size, both overall and within subgroups. Future larger-scale studies are needed to replicate and expand upon these findings and to clarify their relationship with clinical motor outcomes. In addition, future work employing task-based cortico-cortical functional connectivity analyses in source space is needed to confirm whether our interpretation of compensatory/adaptive network strategies in this work truly reflects network-level adaptations. Incorporating trial-by-trial grip force and timing measures would also be valuable to determine how individuals with varying CST injuries regulate motor output to further elucidate the significance of functional connections identified in this study. Together, these efforts would enhance the translational relevance of CMC measures and their potential to inform stroke rehabilitation.

## Conclusion

This work underscores the complexity underlying structure-function relationships in early stroke recovery. These relationships are nuanced, impacted by the type of structural measure and the severity of structural damage. Consideration of specific task demands is also warranted. By employing task-based corticomuscular connectivity measurement, we identified pertinent functional connections that could be leveraged during the design and development of targeted treatments in stroke rehabilitation.

## Data Availability

The datasets presented in this study can be found in online repositories. The names of the repository/repositories and accession number(s) can be found below: Anonymized data available through UNC Dataverse at https://doi.org/10.15139/S3/KPJCQL.
