# Supplementary material for: Associations between structural injury and task-based corticomuscular connectivity after stroke
Source: Front Neurol. 2025 Nov 5;16:1653349. doi: 10.3389/fneur.2025.1653349 (PMC12631422; doi:10.3389/fneur.2025.1653349)
Supplement: Supplementary file 1 [file Table_1.docx]

**Supplementary Material**

**Supplementary Figure 1.** Lesion overlap maps of participants based on corticospinal tract (CST) injury a) mild-moderate subgroup (n=11), b) severe subgroup (n=10), and c) participants unable to perform the task (n=9).

**Supplementary Table 1.** Associations between corticospinal tract (CST) injury and corticomuscular coherence (CMC) between ipsilesional hemisphere and affected upper-extremity before false discovery rate correction in (a) entire cohort (n=21), (b) mild-moderate subgroup (≤50%, n=11) and (c) severe subgroup (>50%, n=10).

(a)

| CST Injury | **Corticomuscular Coherence** | | | **Correlation** | **p-value** |
| --- | --- | --- | --- | --- | --- |
|  | **Region of Interest** | **Muscle** | **Frequency** |  |  |
|  | Premotor | Extensor | Delta | 0.51 | 0.01 |
|  |  | Extensor | High Beta | 0.45 | 0.03 |
|  |  | Flexor | Low Beta | 0.45 | 0.03 |
|  |  | Flexor | Delta | 0.45 | 0.03 |
|  |  | Flexor | High Beta | 0.44 | 0.04 |
|  | M1 | Interossei | Low Beta | 0.45 | 0.03 |

**Supplementary Table 1. Continued**

(b)

| CST Injury | **Corticomuscular Coherence** | | | **Correlation** | **p-value** |
| --- | --- | --- | --- | --- | --- |
|  | **Region of Interest** | **Muscle** | **Frequency** |  |  |
|  | SMA | Extensor | High Beta | 0.83 | 0.001 |
|  |  | Extensor | Low Beta | 0.69 | 0.01 |
|  | Premotor | Extensor | Delta | 0.73 | 0.01 |
|  |  | Flexor | High Beta | 0.66 | 0.02 |
|  |  | Flexor | Low Beta | 0.63 | 0.03 |
|  |  | Extensor | High Beta | 0.63 | 0.03 |
|  | Parietal | Biceps | Low Beta | 0.62 | 0.03 |
|  |  | Biceps | High Beta | 0.62 | 0.03 |
|  |  | Interossei | High Beta | 0.72 | 0.01 |
|  |  | Flexor | Low Beta | 0.70 | 0.01 |
|  |  | Flexor | High Beta | 0.70 | 0.01 |

(c)

| CST Injury | **Corticomuscular Coherence** | | | **Correlation** | **P-value** |
| --- | --- | --- | --- | --- | --- |
|  | **Region of Interest** | **Muscle** | **Frequency** |  |  |
|  | SMA | Extensor | Low Beta | -0.71 | 0.02 |
|  |  | Extensor | Delta | -0.68 | 0.02 |
|  |  | Extensor | High Beta | -0.66 | 0.03 |

**Supplementary Table 2.** Associations between corticospinal tract (CST) integrity and corticomuscular coherence (CMC) between affected extremity and ipsilesional hemisphere in the high integrity (fractional anisotropy>0.62, n=9) group before false discovery rate correction.

| CST Integrity | **Corticomuscular Coherence** | | | **Correlation** | **p-value** |
| --- | --- | --- | --- | --- | --- |
|  | **Region of Interest** | **Muscle** | **Frequency Band** |  |  |
|  | M1 | Biceps | Delta | 0.82 | 0.006 |
|  |  | Interossei | Low Beta | 0.77 | 0.01 |
|  |  | Biceps | Low Beta | 0.94 | 0.0001 |
|  |  | Interossei | High Beta | 0.84 | 0.004 |
|  |  | Biceps | High Beta | 0.79 | 0.009 |
