# Supplementary material for: Associations between structural injury and task-based corticomuscular connectivity after stroke
Source: Front Neurol. 2025 Nov 5;16:1653349. doi: 10.3389/fneur.2025.1653349 (PMC12631422; doi:10.3389/fneur.2025.1653349)
Supplement: Supplementary file 2 [file Table_2.docx]

| **Group** | **Assessment** | **Corticomuscular Coherence** | | | **Correlation** | **p-value** |
| --- | --- | --- | --- | --- | --- | --- |
|  |  | **Region of Interest** | **Muscle** | **Frequency** |  |  |
| Mild-moderate CST Injury | UEFM | SMA | Extensor | High Beta | -0.29 | 0.37 |
|  |  | M1 | Biceps | Low Beta | -0.19 | 0.56 |
|  | ARAT | SMA | Extensor | High Beta | 0.11 | 0.73 |
|  |  | M1 | Biceps | Low Beta | -0.26 | 0.42 |
| Severe CST Injury | UEFM | SMA | Extensor | High Beta | -0.35 | 0.31 |
|  |  | M1 | Biceps | Low Beta | 0.43 | 0.06 |
|  | ARAT | SMA | Extensor | High Beta | -0.10 | 0.76 |
|  |  | M1 | Biceps | Low Beta | 0.51 | 0.13 |
| High CST Integrity | UEFM | SMA | Extensor | High Beta | -0.50 | 0.16 |
|  |  | M1 | Biceps | Low Beta | -0.05 | 0.88 |
|  | ARAT | SMA | Extensor | High Beta | -0.23 | 0.54 |
|  |  | M1 | Biceps | Low Beta | -0.15 | 0.69 |
| Low CST Integrity | UEFM | SMA | Extensor | High Beta | -0.50 | 0.20 |
|  |  | M1 | Biceps | Low Beta | 0.43 | 0.28 |
|  | ARAT | SMA | Extensor | High Beta | -0.03 | 0.93 |
|  |  | M1 | Biceps | Low Beta | 0.61 | 0.10 |

**Supplementary Table 3.** Associations between corticomuscular coherence (CMC) and motor assessments (Upper Extremity Fugl Meyer; UEFM and Action Research Arm Test; ARAT) in subgroups with mild-moderate corticospinal tract (CST) injury (n=11), severe CST injury (n=10), high CST integrity (n=9) and low CST integrity (n=8).

M1, primary motor cortex; SMA, supplementary motor area
