# Supplementary figures and images for: Associations between structural injury and task-based corticomuscular connectivity after stroke
Source: Front Neurol. 2025 Nov 5;16:1653349. doi: 10.3389/fneur.2025.1653349 (PMC12631422; doi:10.3389/fneur.2025.1653349)

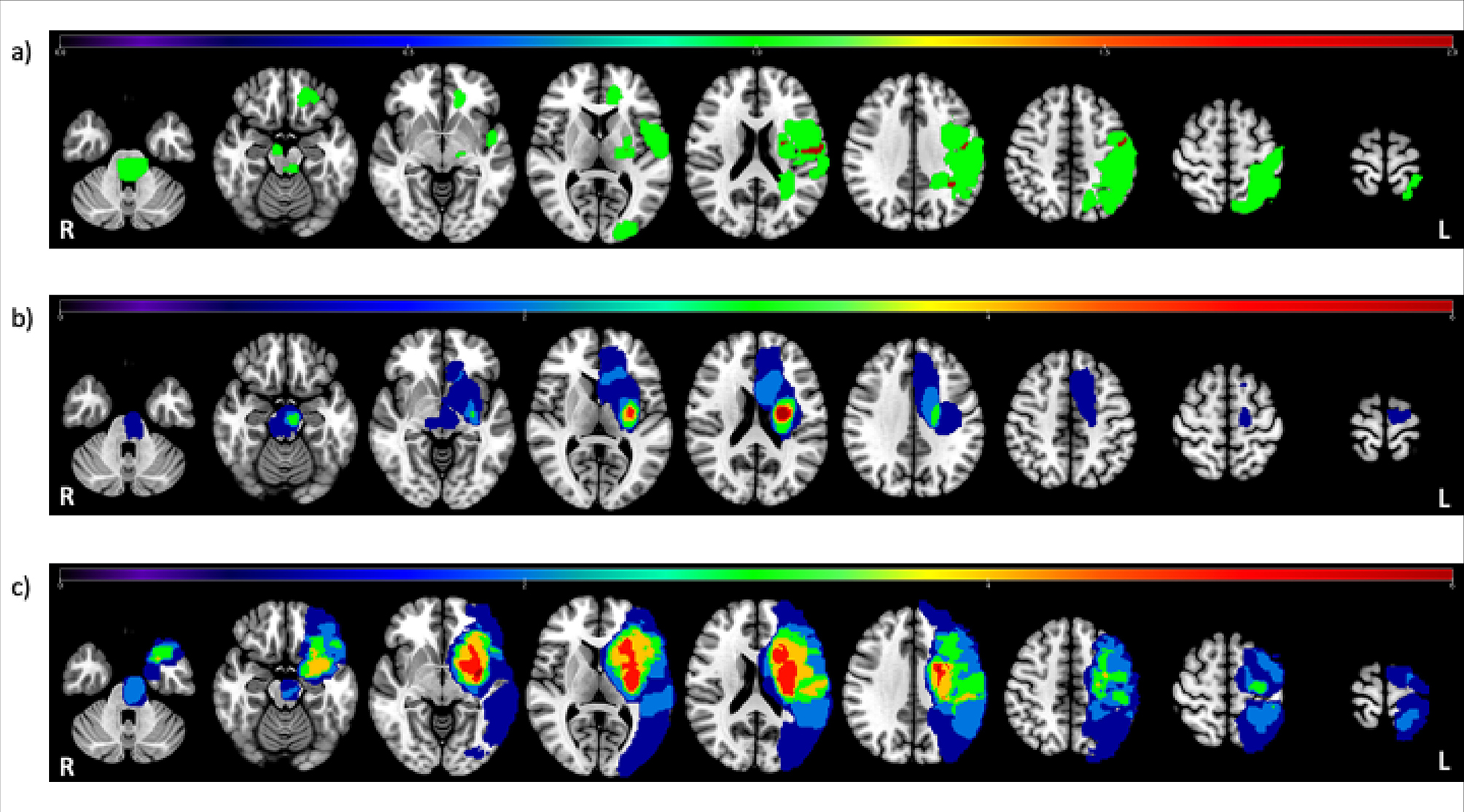

Supplement: Supplementary file 3 [file Image_1.jpeg]
